# Diseases of the Kidney

**Published:** 1906-01-27

**Authors:** 


### Progress in Medicine and Surgery.

### DISEASES OF THE KIDNEY.

(*Continued from page* 273.)

**Tests for Albumen.**—Two methods which are certainly convenient for clinical use have recently been described, and appear to be capable of giving delicate and accurate results. Wish [5] uses a solution of one part pure nitric acid and five parts cold saturated solution of magnesium sulphate. It may be noted that a similar solution has been for many years strongly recommended in the Guy's Hospital Pharmacœpia for ordinary use. Wish uses a small pipette which is lowered into the urine and filled for about one-third of its length. The outside having been wiped, it is dipped for two-thirds its length into the reagent, which is then drawn into it, and the point of contact of the two fluids observed. A white ring indicates the presence of albumen. Payne uses pure nitric acid, and passes the urine over it as in the ordinary "contact" method. In a small flame he then heats the urine, but not the acid, care being taken not to disturb the line of contact. The urine is thus rendered clear, especially if any turbidity due to urates or phosphates was present, and small amounts of albumen not demonstrable in cold urine become apparent after a few minutes. Turbidity due to the presence of resins will disappear on the addition of a few drops of alcohol.

**Multiple Myeloma.**—It is now a recognised fact that cases presenting bone softening in adults may be divided into two classes—mollities ossium and multiple myeloma. The latter condition is often associated with the presence of albumose in the urine, recognised by the fact that it is precipitated by mineral acids and that the coagulum is dissolved by heat and reappears on cooling. Collins,[7] who reports a case where the characteristic albumose was not present, draws attention to the error which may occur if the absence of the Bence Jones albumose is taken as negativing the diagnosis. Only about 50 cases have been reported, so that Collins's carefully written account is a valuable addition to the literature of the subject. The patient was a male 56 years old; his family history was unimportant, but his personal history showed that he had had syphilis, typhoid, rheumatism, and malaria, and was not temperate in alcohol. His illness lasted about three years, beginning with pains in the back and difficulty in micturition. The symptoms gradually became more intense, pain and extreme tenderness of the bones, with progressive emaciation and anæmia being the prominent symptoms. The urine never showed anything but a trace of serum albumen and a few casts, and the blood showed decrease in the red cells and hæmoglobin, with but little disturbance of the normal colour index. The remaining symptoms were due to an enlarged prostate, chronic interstitial nephritis, and some pulmonary fibrosis, with slight hypertrophy and degeneration of the heart muscle. The bones and medulla were occupied by growths, the exact nature of which seems uncertain. Myeloid cells and small round and spindle-shaped cells were seen, so that the case presented some resemblance both to sarcoma and myeloma. It was, however, classed as an example of the latter condition. As Collins remarks, the interest lies in the fact that the patient was under observation throughout the entire illness, and that at no time was any albumose found in the urine.

[5] Med. Rec., April 22, 1905, p. 639. [6] Ibid., April 8, 1905, p. 538. [7] Ibid., April 29, 1905, p. 641.

